# Effect of stretch on conduction in myelinated nerve due to wrist movement: An experimental and analytical study

**DOI:** 10.1371/journal.pone.0333925

**Published:** 2025-10-10

**Authors:** Sabrina Sharmin, Mohammad Abu Sayem Karal, Zaid Bin Mahbub, Khondkar Siddique-e Rabbani

**Affiliations:** 1 Department of Physics, Bangladesh University of Engineering and Technology, Dhaka, Bangladesh; 2 Department of Arts and Science, Ahsanullah University of Science and Technology, Dhaka, Bangladesh; 3 Department of Mathematics and Physics, North South University, Dhaka, Bangladesh; 4 Department of Biomedical Physics and Technology, University of Dhaka, Dhaka, Bangladesh; Università degli Studi di Milano: Universita degli Studi di Milano, ITALY

## Abstract

Based on related measurements by others, an earlier publication suggested increased nerve conduction velocity (NCV) with stretch in myelinated fibers, an anomaly based on existing knowledge, and hypothesized that widening of narrow zigzag gaps between structures of interdigitated Schwann cell processes at the node affected saltatory conduction to produce this increased NCV. A new nodal resistance *R*_*ne*_ between the axonal membrane and extracellular fluid was introduced into the century old cable theory. Later, a direct and careful measurement of ulnar NCV across a 10 cm segment around the elbow by another publication appeared to support the suggestion of increased NCV with stretch. However, in order to eliminate the possibility of slacks of ulnar nerve in the upper arm affecting the measurements, the present work was taken up on a shorter 5 cm segment which again supported the suggestion, increasing confidence in the *R*_*ne*_ hypothesis. Furthermore, wrist flexion or extension was also observed to affect the ulnar NCV at the elbow to some extent, revealing a new phenomenon. While attempting to formulate an analytical treatment of *R*_*ne*_, the earlier work found it very challenging as the physical structure was extremely complex. Proposing an alternative physical model to simulate the variation in *R*_*ne*_ suggested earlier, the current study presents an analytical treatment that relates *R*_*ne*_ and a corresponding effective resistivity value to increases in stretch, and relates these quantitatively to stretch values based on the measured values of NCV. This then provided the basis of a quantitative analysis which could be useful for future research. While appreciating that other microstructures in the node at or near the axonal membrane may also contribute to the observed anomaly, but lack of direct experimental evidence related to nerve stretch tends to weigh more on the *R*_*ne*_ hypothesis in explaining the anomaly.

## 1. Introduction

An instantaneous change in Conduction Velocity (CV) of nerve fibres was reported earlier by head bending experiments [1–3] through changes observed in ‘Distribution of F-Latency (DFL)’, a new nerve conduction parameter [4]. This led to a search of similar experimental findings involving physical manipulations of nerves by Rabbani [5] which revealed a systematic and reversible increase in ulnar nerve conduction velocity (NCV) across a 10 cm region around the elbow due to elbow flexion [6,7]. However, both these latter author groups suggested that this increase was due to measurement errors because of the sliding movements of the nerves. Giving plausible arguments, it was suggested [5] that elbow flexion caused stretching of nerve fibres at the elbow and this should be the major factor causing the systematic increase in NCV in the above experimental findings. This paper also pointed out that stretching would cause a reduction in diameter of the nerve fibres, for which it provided support from experimental findings of other workers [8] who had measured diameter of ulnar nerve at elbow under flexion using ultrasound techniques. Rabbani [5] suggested that this then points to an anomaly with existing knowledge of neural conduction, which states that CV of a fibre depends on the axonal diameter, higher for larger diameters [9]. It was also pointed out [5] that only myelinated nerve fibres contribute to the above NCV measurements carried out using surface electrodes [10] and possibly some mechanisms, unknown till then, caused this anomaly.

**Fig 1:**
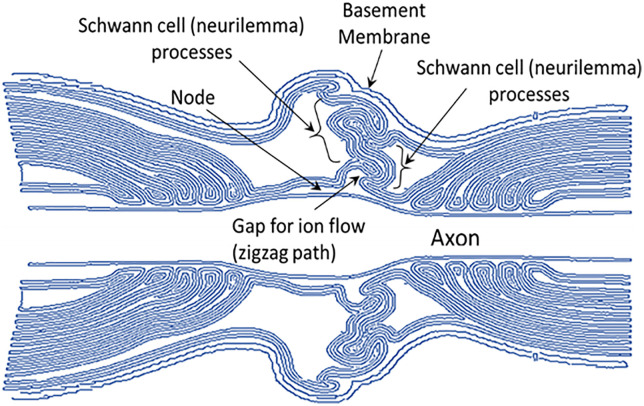
A schematic model of interdigitated Schwann cell (neurilemma) processes at the node of a myelinated nerve fibre, redrawn based on the images and schematic diagrams available [11–13]. A very narrow zigzag gap obstructs ion flow between the outside of the axon and the extracellular space, necessary for saltatory conduction which Rabbani [5] modeled as resistance *R*_ne_, suggesting that this dominates the time delay for propagation of action potential in a myelinated nerve fibre.

Rabbani [5] explained this anomaly based on electron microscopic images of the node of Ranvier and their simplified visualization models. Based on the detailed descriptions and visualization models [11–13], a schematic model of the nodal microstructures is redrawn and shown in Fig 1 showing interdigitated Schwann cell processes (neurilemma) from the two sides of a node, leaving a very narrow and zigzag gap between them.

It may be mentioned that many descriptions of the nodal junction based on electron micrographs do not show these upper structures of neurilemma as they focus on the region very close to the axonal membrane at the node only [14]. Again some descriptions present the neurilemma as continuous at the node [15], without mentioning the presence of a narrow gap. The gaps might not be visible due to the lower resolutions of imaging, which might have led to such conclusions.

These observations led Rabbani [5] to suggest that the interdigitated Schwann cell (neurilemma) processes from the two sides of a node are brought very close in a relaxed state, leaving a zigzag gap of the order of a few tens of nm between them, due to the elastic pull of the endoneurium. The ion flow necessary for the saltatory conduction has to pass through this very narrow zigzag path. In the original published paper, the author mistakenly mentioned these processes as those of *myelin sheaths*, which he realized recently and sent a corrigendum to the journal publishing the original paper [16]. The name should be retained as *Schwann cell processes,* which will be used in all further descriptions and discussions in this paper while referring to results of this work [5] where *myelin sheath* was used before.

**Fig 2:**
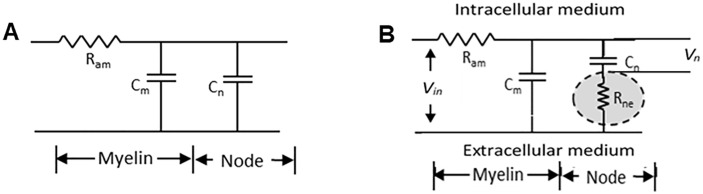
Simplified electrical model of a myelinated nerve fibre. (A) Traditional model (B) Rabbani’s proposed model [5].

Rabbani [5] argued that the minute gap between the interdigitated Schwann cell processes should pose considerable resistance to ionic flow between the outside of the nodal membrane and the endoneurial fluid (extracellular fluid), needed for the saltatory conduction, and that this would dominate the time delay contributing to the CV in myelinated nerve fibres, much more than that due to axonal resistance, as was held earlier (typical axonal diameters are of the order of a few micrometers, a few hundred times to almost a thousand times the gap between the Schwann cell processes at the node). The author [5] modified the century old cable theory [17] introducing a new resistance between the outside of the axonal membrane at the node and the extracellular fluid following the above description and named it as the nodal resistance *R*_*ne*_, which is described further below with the help of Fig 2.

In classical neuroscience it is well established that CV is higher for nerve fibres with larger diameters [18,19], and this aspect was integrated into the cable theory of myelinated nerve fibres where the conduction mechanism was thought to depend on the axonal resistance and respective membrane capacitances [9]. A simplified form of the traditional model for myelinated nerve fibres is shown in Fig 2A based on which a time constant for delay in the propagation of an action potential is given as,


$$\tau_{m}\;=R_{am}\;\;(C_{m}\;+\;C_{n})$$ (1)

Here *R*_*am*_ represents the axonal resistance between two consecutive nodes while *C*_*m*_ and *C*_*n*_ represent the respective capacitances of the internodal region covered by myelin sheath and that of the axonal membrane at the exposed nodal region.

The CV would be inversely related to the time constant, meaning that higher the time constant, lower will be the value of CV, and vice versa. For an axon with a smaller diameter, the axonal resistance *R*_*am*_ would be higher (varying as the inverse square of the diameter), while the capacitances would be lower (varying linearly with the diameter), giving a net increased time constant *τ*_*m*_ and a consequent decreased CV. Of course, this representation would hold good for myelinated nerve fibres with different diameters in relaxed conditions. For unmyelinated nerve fibres, a lumped circuit model will give a similar outcome, i.e., smaller value of CV for a fibre with smaller diameter.

**Fig 3:**
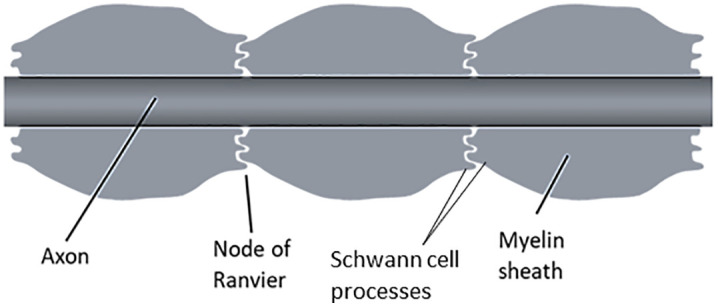
Illustration of a two-dimensional cross-sectional view of a myelinated nerve fibre showing interdigitated Schwann cell processes from both sides of a node of Ranvier [5].


In order to introduce the effect of the minute gap between the Schwann cell processes a modification to the cable theory was proposed [5] as shown in Fig 2B. Here the new nodal resistance *R*_*ne*_ is shown within a shaded ellipse. This is opposed to the clear nodal gap concept existing before, whose impedance to current flow was assumed negligible. The author also proposed a simplified schematic model of a myelinated nerve fibre showing the interdigitated Schwann cell processes at the nodal junction as shown in Fig 3. On stretching, the Schwann cell processes move apart allowing more conducting fluid in the gap reducing *R*_*ne*_ which reduces the time delay for charging and discharging of the nodal membrane capacitance, thus contributing to an increase of CV. This then could explain the observed anomaly mentioned above. This also explains why the changes occur instantaneously. Based on the model shown in Fig 2B, the contribution of *R*_*ne*_ to the time constant for propagation of action potential *τ*_*n*_ was given as (here only the nodal capacitance is involved),

$${\tau \;}_{n}=\;R_{ne}C_{n}$$ (2)

For myelinated nerves the axonal diameters are usually between a few µm to about 20 µm while the gaps between the interdigitated Schwann cell processes in the nodal region would be of the order of tens of nm, as mentioned above, a few hundred to a thousand times less than the axonal diameter. Besides, the path to ionic flow in the nodal gap is not straight, rather it will wind through many interdigitated Schwann cell processes in a zigzag pattern, increasing the resistance to ionic flow. Based on the above scenario, it was suggested [5] that *R*_*ne*_ should be much greater than *R*_*am*_ and that the time constant given by Eq. (2) will be much greater than that given by Eq. (1), i.e.,


$$\tau_{n}\;>>\;\tau_{m}$$ (3)

It means that *τ*_*n*_ would dominate the resultant time constant limiting the propagation of action potentials in myelinated nerve fibres.

Here the nodal membrane capacitance *C*_*n*_ is expected to change slightly on stretching, tending to influence the time delay, however, the rate of decrease in *R*_*ne*_ is expected to be much greater, dominating the overall outcome. Therefore, there will be an overall decrease of time delay and a corresponding increase in CV. As soon as the stretching force is released, the interdigitated Schwann cell processes close in because of the elastic pull of the endoneurium, and so the CV reverts back to original value immediately. This explains the immediate reversal of the measured NCV on removal of the stretching force. Expressing the newly introduced resistance *R*_*ne*_ in terms of an effective path length *l*_*r*_ for the zig-zag path for the ions to flow between the narrow interdigitated protruding parts of the myelin sheath, it was shown [5] that:


$$CV\;\propto \;t_{n}\;/\;l_{r}$$ (4)

Here *t*_*n*_ is the axonal membrane thickness at the node. It was argued that *t*_*n*_ would decrease linearly with stretch while the path length *l*_*r*_ would decrease much more sharply as the interdigitated lips of the Schwann cell processes are pulled apart on stretching. Besides, it will affect the ionic path two dimensionally over a circular region, so the reduction effect will be very high. Therefore, the decrease of *l*_*r*_ with stretch would dominate and consequently CV would increase with stretch, as observed. Again, the CV would restore to the original value when the stretching force is withdrawn. This then explained the experimental observations and removed the anomaly for myelinated nerve fibres on stretch as claimed by the author [5].

However, it was needed to critically evaluate the above hypothesis and the suggested models. Earlier experimental studies [6,7] reported a systematic increase in ulnar NCV in a 10 cm nerve segment at elbow (at 0° elbow flexion) with angle of flexion, but they had thought that this increase could be due to measurement errors as the nerve may slide during measurements as mentioned before, and detailed records of segment lengths as measured were not available. Therefore, the above experimental study was repeated to investigate the effect of elbow flexion on ulnar NCV quantitatively, measuring the lengths of the nerve segment of the ‘above elbow-below elbow’ (AE-BE) segment as accurately as possible from the outside of the skin [20]. These length values were then used to provide magnitudes of stretch following the arguments put forward earlier [5] which says that since the ulnar nerve passes around a rigid bone structure at the medial epicondyle, it is subjected to stretch on flexion [21]. A linear relationship between nerve stretch and angle of elbow flexion was obtained with R^2^ = 0.9881. Following this, a linear relationship was obtained between nerve stretch and the change in NCV. For a nerve stretch of about 31% the ulnar NCV was found to increase by about 19% with R^2^ = 0.9884. The placement of electrodes was made such that at 0°elbow flexion, the measurement covered a distance of 6 cm in the proximal direction from the medial epicondyle for an AE-BE segment length of 10 cm, leaving 4 cm in the distal direction. It is possible that some error may occur at low angles of flexion due to slackness of the nerve trunk in the upper arm [22,23]. However, the contribution of the slack should be small at large angles of flexion.

Considering the above issues, it was felt that if the AE-BE segment length is kept very close to the medial epicondyle, within a shorter segment length, the effects of slacks in the upper arm will be minimized and this was one of the objectives of the present work. Furthermore, wrist extension and flexion may also stretch the ulnar nerve distal to elbow [23]. In the previous measurements [20], this aspect was not taken into account, so it may be worthwhile to study the effect of wrist flexing movement on the ulnar NCV at the elbow.

As the experimental observations appeared to support the proposed mechanism [5], the authors tried to develop further quantitative analyses to obtain relevant mathematical parameters under stretch for myelinated nerve fibres. While attempting this on the basis of Eq. (4), it appeared that further analysis involving the variation of the path length *l*_*r*_ with stretch would be extremely difficult as detailed information of the nodal gap region between the interdigitated lips of Schwann cell processes is still unknown and may be complex. In order to break this impasse, the present work takes an altogether different approach for an analytical study which is detailed below and may be the basis of further quantitative analyses in the future.

## 2. Methods

This section is divided into two parts. The first subsection presents the experimental study while the second subsection presents the new analytical study, mentioned above.

### 2.1. Experimental study

The present experimental work involved two objectives and the methods undertaken are described below.

#### 2.1.1. Minimising the effect of nerve slack in the upper arm.

The previous measurement for NCV of ulnar nerve around the elbow [20] was repeated, but within a smaller AE-BE segment length of 5 cm instead of the previous 10 cm, with the proximal stimulation point at only 2.5 cm proximal to the medial epicondyle as against 6 cm in the previous study. With this smaller segment length, the effects of slacks in the upper arm was expected to be minimized, and the measured NCVs was expected to truly correspond to magnitudes of stretch in the nerve segment, and this was one of the objectives of the present work. The increase in length that was measured between the marked stimulation points proximal and distal to the medial epicondyle for different angles of flexion (with reference to that at 0° flexion) could be reliably interpreted as the amount of stretch produced in the ulnar nerve within that segment. Again, it was argued that, even if there was a small error in measuring the absolute nerve lengths from outside the skin, the error would be negligibly small when the measurements were converted into percentage changes in nerve length.

**Fig 4:**
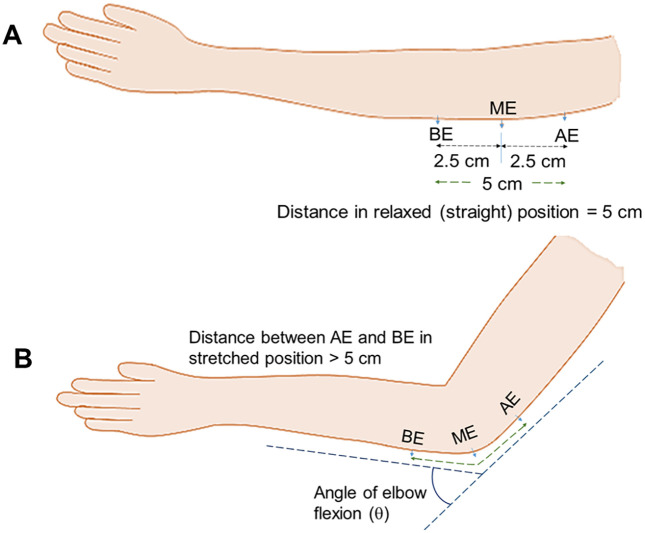
Schematic diagram of elbow position. (A) Relaxed and (B) Flexed positions.

Motor NCVs in the nominal 5 cm elbow segment were experimentally obtained for ulnar nerves of both hands at three specific angles of elbow flexion, 0°, 90° and 135° respectively. Fig 4A shows the schematic diagram of elbow position at a flexing angle of 0° (relaxed state) while Fig 4B shows the same at a flexed position, at an angle *θ*. The positions AE (above elbow), ME (mid elbow – located in reference to the Medial Epicondyle and the Olecranon Process, OP) and BE (below elbow) indicate the positions around the elbow used as references to study the changes in the NCV on elbow flexion. The AE-BE segment was kept constant at 5 cm at the relaxed state or 0° flexion of elbow (Fig 4A), the point AE at 2.5 cm proximal to ME and the point BE at 2.5 cm distal to ME. These positions were marked on the skin using a pen. With the change of angle of flexion, the distances of the AE-ME and BE-ME segments (from the respective pen markings to the OP) increased which were carefully measured. Adding both these segment lengths for each angle of flexion the total length of the AE-BE segment was obtained.

#### 2.1.2. Study on the effect of wrist extension and flexion.

Using the above experimental condition, the effect of wrist extension and flexion on the ulnar NCV in the AE-BE segment for different angles of elbow flexion at were examined.

**Fig 5:**
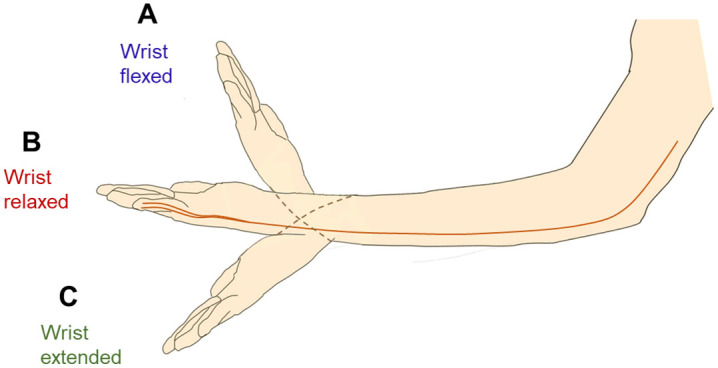
Schematic diagram of wrist position during the study. (A) Flexed (B) Relaxed (C) Extended positions.

Motor NCVs of the ulnar nerves of both hands in the elbow segment were experimentally obtained for three different angular positions of wrist: flexed, relaxed and extended, as shown in Fig 5. Each of these measurements were carried out for the above three specific angles of elbow flexion, 0°, 90°, and 135° respectively. It was expected that the flexed state of the wrist would contribute to a slack in the ulnar nerve compared to that in the relaxed position (0°). On the other hand, the nerve is expected to be in a stretched state in its extended position as shown in Fig 5. Through these measurements, it would be possible to find out how these changes in wrist positions affect the NCV at the elbow region.

#### 2.1.3. Study details.

For this study on human subjects, necessary ethical approval was obtained from Bangladesh Medical Research Council ( BMRC ) (Registration No. 368 17 12 2020). All procedures were performed in accordance with the ethical standards of the National Research Ethics Committee ( NREC ) of BMRC . No minor subjects were involved. The subjects volunteered to participate in the study with informed written consent, according to the protocol of BMRC . The duration of the study was from 11 January 2023 to 25 March 2023 .

**Fig 6:**
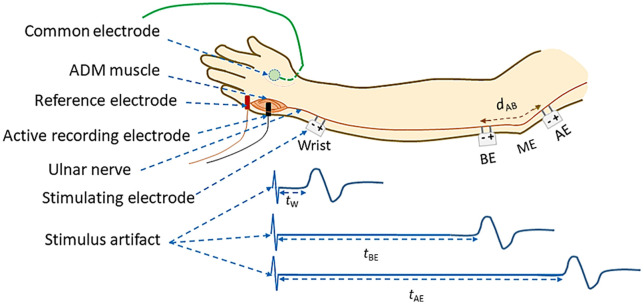
Schematic diagram of electrode arrangement for the measurement of NCV of the ulnar nerve between different nerve segments.

A total of 12 nerves from the left hands of healthy subjects (6 males and 6 female) of mean age 30 years, without any diagnosed neurological disorder, were examined. For measurement of NCV , a Nicolet EDX system ( Natus Neurology, Middleton, WI USA) was used. A schematic diagram of electrode arrangement for the measurement of NCV is presented in Fig 6. The active recording electrode was placed on the prominent part of the abductor digiti minimi (ADM) muscle. The reference electrode was placed over the hypothenar tendon at the metacarpophalangeal (MCP) joint of the fifth digit. The inter-electrode distance was kept constant at 4 cm from center to center. The common or neutral electrode was placed on the dorsum of the hand. Electrical stimulation was applied at three positions: i) wrist (W), about 7 cm proximal from the active recording electrode, ii) below elbow (BE), and iii) above elbow (AE). Each of these produced corresponding compound muscle action potentials (CMAP) as shown schematically in Fig 6. As mentioned before, the distance between BE and AE was chosen to be 5 cm at 0° angular position of elbow (Fig 4A), which was taken to be the relaxed condition.

In Fig 6, the latency value *t*w corresponds to the time between the stimulus applied to the wrist and the onset of the CMAP as recorded from the ADM muscle of the palm. Similarly, *t*BE and *t*AE are the corresponding latencies obtained due to the stimulus applied at BE and AE respectively. The latency *t*w includes the uncertain latency at the neuro-muscular junction which was subtracted from *t*BE and *t*AE to obtain the corresponding NCV of the nerve segments BE-W and AE-W respectively, following standard procedures. The NCV for the AE-BE segment around the elbow, *v*AB was determined through a subtraction of the above latency values, *t*BE and *t*AE and the corresponding measured distances between two stimulating sites, AE and BE, according to the formulae given below:


$$v_{AB}=\frac{d_{AB}}{t_{AB}}$$ (5)

Where *d*AB is the length of the AE-BE segment and *t*AB is the latency difference between the AE and BE sites.

#### 2.1.4. Statistical analysis.

The experimental results were analyzed to determine whether the NCVs indeed increased in a systematic way with angle of flexion, or if these varied randomly. For this purpose, Page’s Trend test [24] was performed to test the statistical significance of the trend of change in velocity, *v*AB. According to this test the Null Hypothesis and the Systematic Incremental hypothesis were set as follows, the subscripts of the velocity symbol, *v*, indicating the angles of elbow flexion.

*Null Hypothesis, H0:* *v*0 = *v*90 = *v*135,

*Systematic Incremental hypothesis, Hi: v*0 < *v*90 < *v*135.

The test was also performed to observe if there is any systematic trend in the variation of *v*AB with the variation of wrist positions. The corresponding outcomes are discussed in the *Results and observations* section.

### 2.2. Analytical study

This subsection describes the methods taken up for the new analytical approach mentioned before, in order to study the effect of stretch on the change in CV .

**Fig 7:**
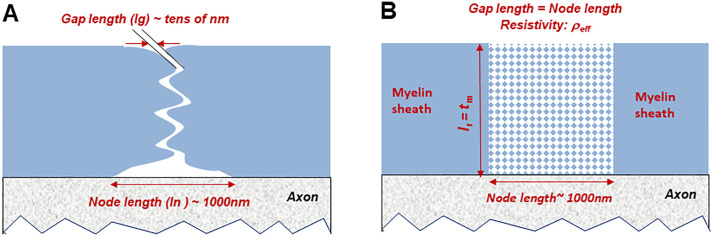
Visualisation of nodal gaps and relationship with new nodal resistance *R*_*ne*_. (A) Nodal gap length *l*_*g*_ between Schwann cell processes are much smaller than node length *l*_*n*_, just outside the axonal membrane. *R*_*ne*_ decreases with nerve stretch that increases *l*_*g*_. (B) Visualisation of nodal gap based on the proposed simplified approach. The nodal gap length is assumed the same as the node length but the nodal gap region is assumed to have a variable resistivity *ρ*_*eff*_, due to a combination of fluid and small insulating particles representing the Schwann cell processes (dotted part). With stretch the fluid volume increases, decreasing *ρ*_*eff*_, thus decreasing *R*_*ne*_, which reverts back to the previous value on removing the stretch.

A pictorial representation of the nodal region in terms of the physical model proposed by Rabbani [5] is shown in Fig 7A. Here the nodal width at the axonal membrane *l*_n_ is much larger than the actual nodal gap *l*_g_ existing between the interdigitated Schwann cell processes, the zigzag path restricting the radially directed ionic flow required for saltatory conduction and contributing to the nodal resistance *R*_*ne*_. Typically, *l*_n_ would be of the order of 1μm (1,000 nm) while *l*_g_ would be of the order of tens of nm, as mentioned before. With stretch, both are expected to increase through the same percentage. As mentioned earlier, the interdigitated Schwann cell processes have complex geometries in 3D, which again would change on stretch and an analysis to determine *R*_*ne*_ based on this physical representation would be very difficult. Therefore, in the present work, an analysis for *R*_*ne*_ has been developed using a hypothetical model to simulate the physical situation functionally, leading to a simplified approach as discussed below.

The new approach assumes a hypothetical model as shown in Fig 7B. Here, the nodal gap is considered the same as the nodal width at the axonal membrane, *l*_n_, all along the width of the myelin sheath. This region is considered to be filled with a conducting fluid, but embedded with small insulating particles (represented by the dotted points in the figure) that simulate the Schwann cell processes from the two sides of the gap. Thus, it is similar to the age-old model of a wide fluid filled nodal gap but with an important difference, that brought about due to the presence of the small insulating particles. In the age-old model one would consider the resistivity of the nodal fluid to be constant whether at rest or under stretched conditions, while in this new model an effective resistivity, *ρ*_eff_ of the nodal gap region is considered, which would vary with stretch. On stretch, the Schwann cell processes move further apart causing an influx of extracellular fluid, thus increasing the fluid volume within the above nodal gap region, but with no change in the number of insulating particles and the volumes they occupy. This decreases the effective resistivity, *ρ*_eff_. On the other hand, on removing the stretch, the extra fluid flows back to the extracellular region, increasing *ρ*_eff_ back to the previous value. This is comparable to the definition of lung impedance where the effective resistivity of the lung region is taken as a combination of inner lung tissue and air volume, the latter changing with the breathing cycle.

Here the direction of ionic current flow within the nodal gap is radial, so that the path length for ion flow *l*_*r*_ would be in the vertical direction as shown in Fig 7B. Thus in the present model, *R*_*ne*_ may be given by,


$$R_{ne}=\frac{\rho_{eff}l_{r}}{A_{av}}$$ (6)

Here *A*_av_ is the average area of the cylindrical nodal gap region expressed approximately as,


$$A_{\text{av}}=X_{av}l_{n}$$ (7)

where *X*_av_ is the average cylindrical peripheral length of the myelin sheath at the node.

Now, it may be seen from Fig 7B that the path length *l*_r_ equals the thickness *t*_m_ of the myelin sheath at the nodal region and Thus replacing *l*_r_ by *t*_m_ in Eq. (6) and using Eq. (7), one gets,


$$R_{ne}=\frac{\rho_{eff}t_{m}}{X_{AV\;}l_{n}}$$ (8)

The detailed analyses and outcomes are presented in the *Analytical study* sub-section below, under the *Results and observations* section.

## 3. Results and observations

### 3.1. Experimental study

#### 3.1.1. Change in NCV with different positions of elbow and wrist.

**Fig 8:**
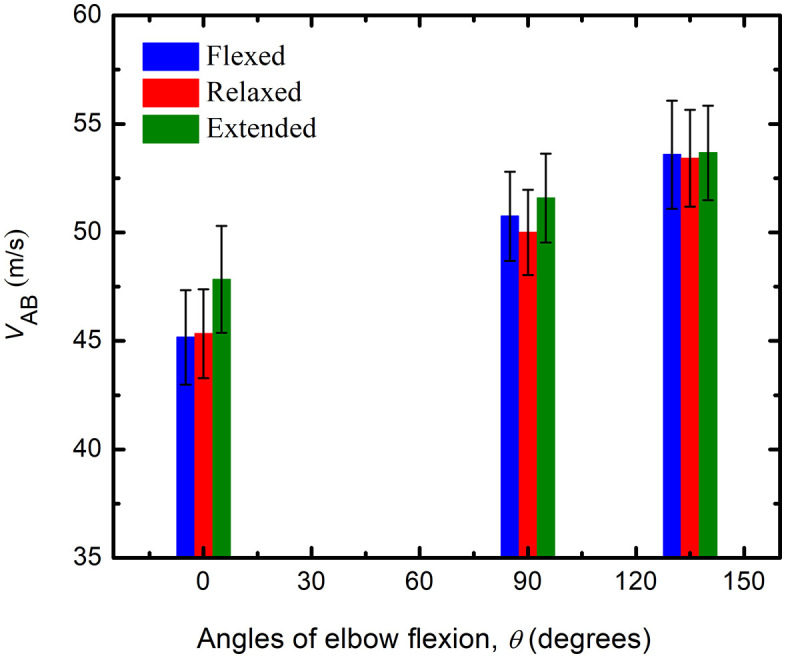
The variation of *v*_AB_ with angles of elbow flexion at different positions of wrist (flexed, relaxed, extended).

The average NCVs for a 5 cm AE-BE segment (*v*_AB_) of all subjects for three different angles of elbow flexions and three wrist positions are presented as bar graphs in Fig 8, where the height of each bar represents the corresponding average *v*_AB_. It is evident from the graphs that, a gradual increase in *v*_AB_ with the angle of elbow flexion is observed for all positions of wrist, which agrees with the results of the previous work, carried out on 10 cm nerve segment [20].

From Fig 8 one can also observe the effect of wrist bending on *v*_AB_, for different angles of elbow flexion. At 0° elbow flexion, with respect to that for the relaxed position of the wrist the NCV is almost the same for the flexed position while it is higher at the extended position. At 90° elbow flexion, with the NCV at the relaxed position of the wrist as the reference, that at flexed position is slightly higher, and is more so for the extended positions, but the change is not as large as observed for 0° elbow flexion. At 135° elbow flexion, the NCV remains almost same for the all the positions of wrist, there does not seem to be any effect of changed wrist positions.

#### 3.1.2. Change in NCV with percentage of nerve stretch.

As mentioned before, for each elbow angle we measured the NCV (*v*_AB_) for the AE-BE segment between the marked stimulation points around the elbow (5 cm at 0^°^ elbow angle, increased for increased angles of flexion) and also calculated the corresponding percentages of stretch corresponding to the increases in the length. The mean *v*_AB_ values together with the corresponding standard errors of mean (± SErr) at different angles of elbow flexion, the corresponding percentages of stretch and for different wrist positions are presented in Table 1. The respective P values using Page’s Trend test are also shown. Their values are much less than the critical value of 0.05 indicating rejection of the null hypothesis for a changing trend with nerve stretch for all positions of wrist. This means that a systematic incremental hypothesis with increased stretch holds for NCV measured over a 5 cm segment at elbow.

**Table 1:** Average values (± SErr) of *v*_AB_ corresponding to different angles of elbow flexion and different percentages of stretch for three positions of wrist. Corresponding P values obtained using Page’s Trend test are also shown.

Angle of elbow flexion (deg)	% Stretch (Approx)	Wrist flexed		Wrist relaxed		Wrist extended	
		*v*_AB_ (m/s) (mean ± SErr)	P value for trend	*v*_AB_ (m/s)	P value for trend	*v*_AB_ (m/s)	P value for trend
0	Reference	45.2 ± 2.2	0.0004	45.3 ± 2.0	0.0002	47.8 ± 2.5	0.0011
90	22.8	50.8 ± 2.0		50.0 ± 1.9		51.6 ± 2.0	
135	36.2	53.6 ± 2.5		53.4 ± .2.2		53.7 ± 2.2	

It needs to be noted that this study gives *v*_AB_ for a 5 cm nerve segment around the elbow at relaxed position, while previous experiments by [6,7,20] performed the measurements on a 10 cm nerve segments. The shorter segment of 5 cm, with 2.5 cm on each side of the Medial Epicondyle is expected to be less affected by any slackness of the ulnar nerve in the upper arm.

**Fig 9:**
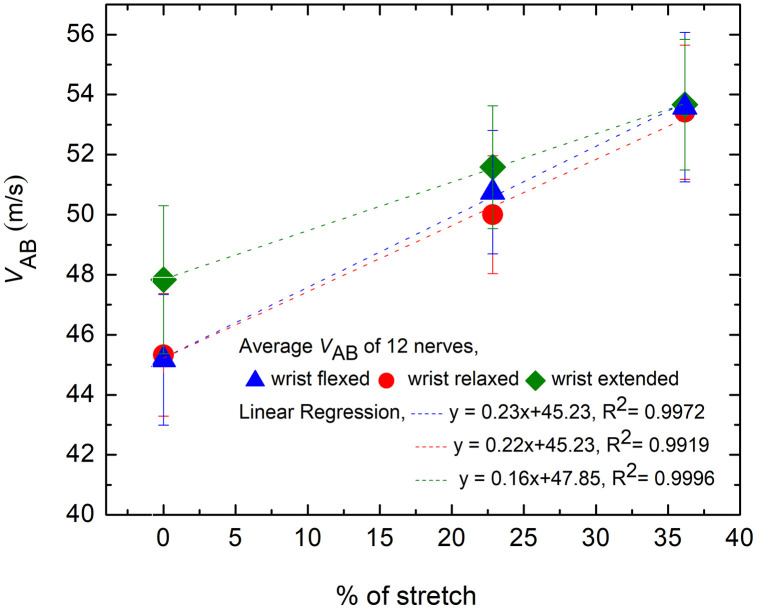
The change of *v*_AB_ plotted against the percentage of nerve stretch due to elbow flexion, with three positions of wrist, the reference of the measured stretch being the corresponding value at 0° angle of elbow flexion. Fitted straight lines are also shown.

The NCV in the AE-BE segment, *v*_AB_ is plotted in Fig 9 against the percentage of nerve stretch as measured, for all three positions of the wrist. The fitted straight line graph with equation and the R^2^ values are also shown. The trend line equations in Fig 9 have R^2^ values of 0.9972, 0.9919 and 0.9996 for flexed, relaxed and extended position of wrist, respectively, indicating that each has a good linear fit. The rate of change of *v*_AB_ is about 0.23, 0.22 and 0.16 m/s per unit percentage stretch of nerve, for the flexed, relaxed and extended position of wrist, respectively.

#### 3.1.3. The percentage changes in NCV with percentage of nerve stretch.

The percentage changes in the motor NCV of the ulnar nerve at the elbow (*v*_AB_) with reference to that at 0% stretch, are shown in Table 2.

**Table 2:** Percentage increase in segmental conduction velocity, *v*_AB_ corresponding to percentage of nerve stretch (AE-BE segment length at 0° flexion taken as reference).

% Nerve stretch (Approx.)	% Increase in *v*_AB_ (referenced to corresponding values at 0° elbow flexion)		
	Wrist flexed	Wrist relaxed	Wrist extended
Reference	0	0	0
22.8	12.3	10.3	7.8
36.2	18.6	17.8	12.2

**Fig 10:**
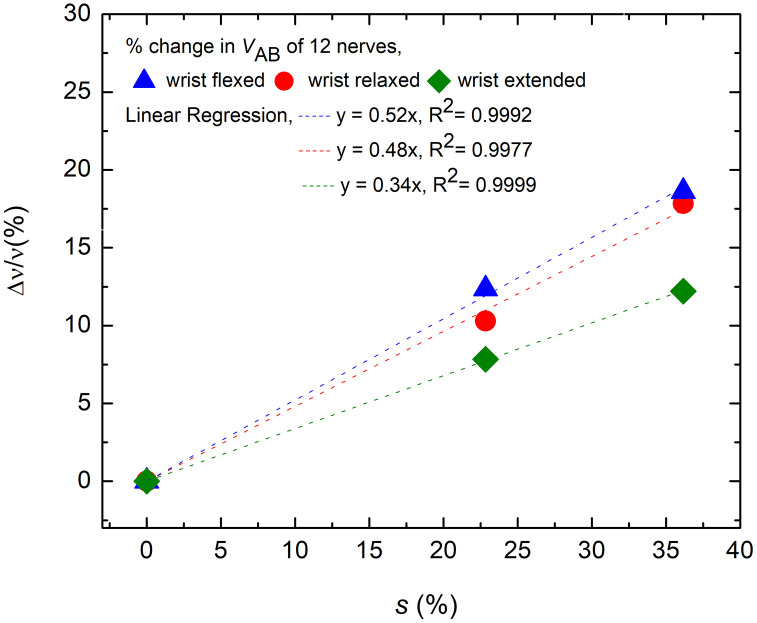
The percentage change of *v*_AB_ for the flexed, relaxed and extended position of wrist, plotted against the percentage of nerve stretch due to elbow flexion, the reference of the former being the corresponding value at 0% stretch (0°elbow flexion).

Fig 10 presents the percentage changes in *v*_AB_ against the percentage of nerve stretch due to elbow flexion for the flexed, relaxed and extended position of wrist. The linear trend lines have R^2^ value of 0.9992, 0.9977 and 0.9999 for flexed, relaxed and extended position of wrist respectively. The corresponding rates of change in the percentage change in *v*_AB_ are about 0.52%, 0.48%and 0.34% per unit percentage stretch of nerve respectively.

#### 3.1.4. Change in NCV due to wrist position.

The Page’s Trend test was also performed to see whether there was any significant trend in the variation of *v*AB due to the wrist position and are presented in Table 3. The P values obtained for all positions of wrist are greater than the critical value of 0.05, indicating no significant trend of variation of *v*AB with wrist position.

**Table 3:** Average values (± SErr) of *v*_AB_ corresponding to different positions of wrist and different angles of flexion of elbow. Corresponding P values obtained using Page’s Trend test are also shown.

Position of wrist	0° elbow flexion		90° elbow flexion		135° elbow flexion	
	*v*_AB_ (m/s)	P value	*v*_AB_ (m/s)	P value	*v*_AB_ (m/s)	P value
Flexed	45.2 ± 2.2	0.1530	50.8 ± 2.0	0.3074	53.6 ± 2.5	0.4142
Relaxed	45.3 ± 2.0		50.0 ± 1.9		53.4 ± .2.2	
Extended	47.8 ± 2.5		51.6 ± 2.0		53.7 ± 2.2	

However, considering only the relaxed and extended positions of the wrist, it may be seen that for 0^°^ and 90^°^ angles of elbow flexion, some increase could be noted. These are presented in Table 4.

**Table 4:** Average values of *v*_AB_ corresponding to different positions of elbow flexion for only relaxed and extended positions of wrist.

Position of wrist	0° elbow flexion		90° elbow flexion		135° elbow flexion	
	*v*_AB_ (m/s)	Increase, %	*v*_AB_ (m/s)	Increase, %	*v*_AB_ (m/s)	Increase, %
Relaxed	45.3	Reference	50	Reference	53.4	Reference
Extended	47.8	5.52	51.6	3.20	53.7	0.56

For 0° and 90° elbow flexion, the *v*AB increased by 5.52% and 3.2% respectively at extended position of wrist with respect to those at the corresponding relaxed positions, while for 135° elbow flexion the increment was very small, only 0.56%.

To see whether the change in NCV for 0° elbow flexion is significant between the relaxed wrist and extended wrist, Page’s Trend test was performed. The P value was obtained at 0.08. Although the change for wrist relaxation and extension is not significant at a P > 0.05, but a P of 0.08 seems to indicate that there is a tendency of change.

### 3.2. *Analytical study*

#### 3.2.1. Development of a basic quantitative model for nerve stretch.

This section presents the analytical study on the effect of stretching on different parameters affecting nerve conduction. Considering the cylindrical axon as a closed system filled with incompressible fluid, the volume of an axonal segment may be expected to be constant on stretching. This gives, for the fractional change in volume,


$$\frac{\Delta V}{V}=\text{0}$$ (9)

This equation forms the basis of estimating the variations in other related parameters. The same incompressibility of volume will be used for the annular cylindrical region of the myelin sheath shown in Fig 7B (solid light blue colour), however, the fingerlike Schwann cell processes, considered as separate particles within the nodal gap region, are not included in this volume. For the proposed analytical model, this incompressibility will not be applicable to the nodal gap region shown dotted in Fig 7B since it is an open system with fluid interchange with the endoneurial region.

In this analysis, the symbol *s* will be used to represent the fractional elongation, i.e.,


$$\frac{\Delta l}{l}=s$$ (10)

which is applicable to any length parameter along the axon (*l*_n_, *l*_g_, etc.).

**Fig 11:**
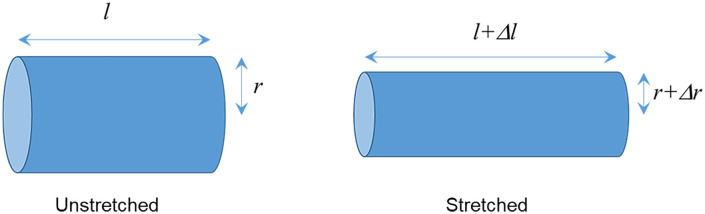
Effect of stretching on a cylindrical structure. Here *Δ*r** is negative.

Now, considering the effect of stretch on a cylindrical volume of length *l* and radius *r,* if a stretching force is applied along its length, *l* will increase. Since the volume does not change, the radius *r* should decrease as shown in Fig 11. However, for mathematical analysis, we consider the changed length and radius as (*l* + Δ*l*) and as (*r + Δ*r**) respectively, keeping in mind that with stretch, Δ*l* would be positive while *Δ*r** would be negative.

Now the volume of the cylindrical region is expressed as,

$V=\pi r^{\text{2}}l$, which will remain unchanged following Eq. (9).

Applying methods of differential calculus for small changes, this gives,

$\frac{\Delta V}{V}=\text{2}\frac{\Delta r}{r}+\frac{\Delta l}{l}\;=\;\text{0}$, from which one obtains,


$$\frac{\Delta r}{r}=\;-\frac{\text{1}}{\text{2}}\frac{\Delta l}{l}\;=\;-\frac{\text{1}}{\text{2}}\;\text{s}$$ (11)

Since CV is inversely proportional to the time constant *τ*, which approximately equals *τ*_*n*_ as given by Eq. (2), it may be expressed as, replacing CV by *v,*


$$v\;\;\propto \;\frac{\text{1}}{R_{ne}C_{n}}$$ (12)

where *C*_*n*_ is the capacitance of the axonal membrane at the node and given by,


$$C_{n}=\;\frac{{\text{2}\epsilon \pi r_{n}l}_{n}}{t_{n}}$$ (13)

ϵ being the permittivity of the axonal membrane, *r*_*n*_ is its average radius at the nodal region with length *l*_*n*_ while *t*_*n*_ is the thickness of the axona*l* membrane a*t* the node.

Now to obtain the value of the fractional variation of C_*n*_, i.e., of $\frac{{\Delta C}_{n}}{C_{n}}$ from Eq. (13), it should be noted that *t*_*n*_ is a direct function of the radius since i*t* is the difference between the external and internal radii of the axonal membrane. Therefore, both *r*_*n*_ and *t*_*n*_ will have the same fractional variation as *t*hat of *r*, which is $-\frac{\text{1}}{\text{2}}s$ [Eq. (11)] and will cancel out when one performs a variational analysis of Eq. 13 leaving only *l*_*n*_ to be considered. Using Eq. (10), one then gets,


$$\frac{{\Delta C}_{n}}{C_{n}}=\frac{{\Delta l}_{n}}{l_{n}}=s$$ (14)

Thus from Eq. (12), the variation of *v* may be expressed as


$$\frac{\Delta v}{v}=-\;\frac{{\Delta R}_{ne}}{R_{ne}}-\;\frac{{\Delta C}_{n}}{C_{n}}\;=-\;\frac{{\Delta R}_{ne}}{R_{ne}}-\;s$$ (15)

From which we obtain the important relationship to express the variation of *R*_*ne*_ with stretch,


$$\frac{{\Delta R}_{ne}}{R_{ne}}=-(\frac{\Delta v}{v}+s)$$ (16)

Here the term $\frac{\Delta v}{v}$ may be obtained from experimental measurements in terms of *s*, which will thereby give $\frac{{\Delta R}_{ne}}{R_{ne}}$ in terms of *s*.

The fractional variation of *ρ*_eff_ with stretch in terms of the fractional variation of $R_{ne}$ can be obtained using Eq. (8) $\left[R_{ne}=\frac{\rho_{eff}t_{m}}{X_{AV\;}l_{n}}\right]$ as follows,


$$\frac{{\Delta R}_{ne}}{R_{ne}}=\;\frac{{\Delta \rho }_{eff}}{\rho_{eff}}+\frac{{\Delta t}_{m}}{t_{m}}-\frac{{\Delta X}_{av}}{X_{av}}-\frac{{\Delta l}_{n}}{l_{n}}$$

$$\text{Rearranging},\;\;\frac{{\Delta \rho }_{eff}}{\rho_{eff}}=\frac{{\Delta R}_{ne}}{R_{ne}}-\frac{{\Delta t}_{m}}{t_{m}}+\frac{{\Delta X}_{av}}{X_{av}}+\frac{{\Delta l}_{n}}{l_{n}}$$ (17)

As argued for *t*_*n*_ above, the fractional variation of *t*_*m*_ should be the same as that for *r* (Eq. 11).

Now *X*_*av*_, the average cylindrical peripheral length of the myelin sheath is directly related to the radius, therefore, fractional variation of *X*_*av*_ will also be the same as for *r*.

So, the part [$-\frac{{\Delta t}_{m}}{t_{m}}+\frac{{\Delta X}_{av}}{X_{av}}$] in Eq. (17) cancels out and using Eq. (10), Eq. (17) becomes,


$$\frac{{\Delta \rho }_{eff}}{\rho_{eff}}=\;\frac{{\Delta R}_{ne}}{R_{ne}}+s$$ (18)

This equation will give the fractional variation of *ρ*_*eff*_ when the fractional variation of *R*_*ne*_ is known from experimental observations based on Eq. (16).

#### 3.2.2. Application of experimental values to the analytical model.

This section presents the quantitative values of the percentage changes of *R*_*ne*_ and *ρ*_*eff*_ as obtained by introducing experimentally observed values to the analytical model developed above.

From the trend line equation in Fig 9 for the relaxed state of wrist, the conduction velocity varies with stretch as:


$$\frac{\Delta v}{v}=\text{0}.\text{4}\text{8}\;s$$ (19)

Rounding off the value (0.48 ≈ 0.5) one gets,


$$\frac{\Delta v}{v}=\text{0}.\text{5}\;s=\text{0}.\text{5}\frac{\Delta l}{l}$$ (20)

This means that for 1% increase in length (i.e., **s* *= 1%), *v* increases by 0.5%.

Then Eq. (16) gives,


$$\frac{{\Delta R}_{ne}}{R_{ne}}=-\text{1}.\text{5}\;s$$ (21)

Which means that for 1% increase in length (i.e., **s* *= 1%), *R*_*ne*_ decreases by 1.5%.

Then Eqs. (18) and (21) gives,


$$\frac{{\Delta \rho }_{eff}}{\rho_{eff}}=\;-\text{0}.\text{5}\;s$$ (22)

Which means that for 1% increase in length (i.e., **s* *= 1%), *ρ*_*eff*_ decreases by 0.5%.

**Fig 12:**
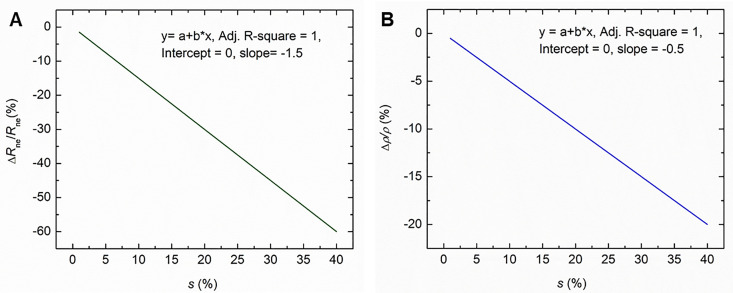
The percentage change in (A) *R*_*ne*_ and (B) *ρ*_*eff*_ with percentage of stretch *s* based on Eqs. (21) and(22) respectively.

Eqs. (21) and (22) suggest linear relationships between the variations in *R*_*ne*_ and *ρ*_*eff*_ with the percentage of nerve stretch. The variations of these two parameters, *R*_*ne*_ and *ρ*_*eff*_ (shown as *ρ* only) against percentage of stretch *s* are shown graphically in Fig 12A and 12B respectively.

Thus based on the above analytical approach and experimental results, it may be summarized that for 1% stretch of nerve, the conduction velocity, *v* increases by 0.5%, *R*_*ne*_ decreases by 1.5% and *ρ*_*eff*_ decreases by 0.5%.

## 4. Discussion and conclusions

The present work was taken up to study a new mechanism of nerve conduction in myelinated nerves proposed by a recent work [5] which suggested that interdigitated Schwann cell processes restrict ion flow between axonal membrane and the extracellular space that are necessary for saltatory conduction and modeled this as an electrical resistance (*R*_*ne*_). It was suggested that at relaxed condition of the nerve, *R*_*ne*_ is very high and dominates the time delay for propagation of action potential. With stretching of the nerve, *R*_*ne*_ decreases sharply, reducing the above time delay for propagation, contributing to an increased CV as observed experimentally. Of course, Rabbani [5] had termed this as an anomaly since it contradicted predictions based on existing concepts and theories of nerve conduction which would suggest a decreased CV with stretch. This is because the fibre diameter is reduced with stretch and existing theories for the dependence of CV on fibre diameter would suggest a decreased CV. In fact, Electron Micrographic and Electron Tomographic studies reveal that Schwann cell (neurilemma) processes and paranodal loops in peripheral nerves form interdigitated, finger-like projections at the node of Ranvier [11–13]. Structural evidence from electron microscopy and molecular studies suggests that paranodal loops and glial microvilli tightly interface with the axolemma, creating a complex geometry at the node [25,26]. While the specific configuration of these structures during stretch remains unconfirmed, they provide a plausible anatomical basis for hypothesized changes in nodal resistance in addition to those suggested earlier based on the narrow gaps between the outer Schwann cell (neurilemma) processes [5].

An earlier work by the authors of the present work [20] provided support to the new mechanism, however, there were points which needed to be looked into more critically. The present study also attempted to develop an analytical model for quantifying this new mechanism, assuming it holds good. Therefore, this present study is segregated into two major parts:

(i) An experimental study of ulnar NCV at elbow region to indicate if removal of slacks of the nerve in the upper arm contributed to an observed increase in NCV with elbow flexion, as suggested by some earlier workers [22,23], or whether it is the new mechanism mentioned above [5] that should be the major contributor. Additional investigations to study the effect of wrist flexion or extension on ulnar NCV at elbow region were also performed, which no one performed before.
(ii) As findings of the above study went in favour of the new mechanism, the second study involved developing an analytical tool based on a simplified physical and electrical model of the node of Ranvier to obtain quantitative parameters that may be used to evaluate changes in nodal resistance and resistivity with changes in nerve stretch. These could form the basis for further studies in the future using the new mechanism.

In the first experimental study, the following two aspects were examined.

(a) To identify whether it is the removal of slacks in the upper arm [22,23] or whether it is the new mechanism proposed by Rabbani [5] that contributed to an observed increase in NCV with elbow flexion [6,7,20], the present experimental study was carried out on a 5 cm segment of ulnar NCV at the elbow for different angles of flexion and the results were compared with those of a similar study performed earlier on a 10 cm segment [20] by the same authors. On stretch due to elbow flexion, the 10 cm segment included more of the upper arm segment (6 cm above medial epicondyle) while the 5 cm included less of the upper arm segment (2.5 cm above medial epicondyle). If the trends and rates of change of NCV with stretch were identical, this would act as a stronger support to the new nerve conduction mechanism proposed by Rabbani [5]. On the other hand, if the trends and rates of change of NCV were significantly different this would mean the presence of some other mechanisms and removal of the slack in the ulnar nerve in the upper arm could be considered if the changes were in an appropriate direction.
(b) Secondly, it examined the effect of wrist bending on the measured NCV at the elbow segment, for different angles of elbow flexion, with the assumption that some of these wrist positions (flexion, relaxed, extended) may further stretch the ulnar nerve at the elbow. This is also an interesting study performed by no one before.

The second study, on developing an analytical tool for quantitative evaluation of the effect of stretch on NCV assuming that the new nerve conduction mechanism [5] holds good was divided into two parts as well. These are:

(a) Proposal of a new approach to simulate the conditions at the node of Ranvier on stretch based on which a simple analytical tool may be developed to express the relevant parameters at the nodal region quantitatively that could be used for future research.
(b) Obtaining changes in nodal resistance and resistivity on stretch, based on this new analytical model and the experimental findings from this study.

The current experimental study on a 5 cm elbow segment of ulnar nerve (of which 2.5 cm was above the medial epicondyle) confirmed that the NCV increased with the angle of elbow flexion for all three wrist positions—flexed, relaxed, and extended—as Figs 9–10, and Table 1 illustrate. However, the rate of increase for relaxed wrist position, which was also the condition for the earlier experiment on 10 cm segments of nerve (of which 6 cm was above the medial epicondyle) [20], was slightly reduced. In the current study the variation of NCV value was approximately 0.5% for 1% stretch for a relaxed wrist position as depicted in Fig 10. The corresponding value was 0.6% in the previous study on 10 cm elbow segments [20], marginally higher than the present value. Therefore, this lends a strong support to the suggestion that the NCV of myelinated nerve fibres increase with stretch and to the new mechanism for increase in CV as proposed [5]. The extra small increment in the previous study on a 10 cm nerve segment [20] could indicate a small contribution from straightening of the slacks in the upper arm since this measurement involves a larger segment of the nerve in the upper arm. Therefore, the measured increases in NCV for a 5 cm segment at the elbow could be almost entirely attributed to nerve stretch.

The impact of wrist bending on the NCV of the elbow portion of the ulnar nerve was examined in the second investigation. As previously stated, this was based on the assumption that some wrist angles produced further stretch of the ulnar nerve.

From the bar graph in Fig 8 and from Table 4 it may be seen that, at 0° angle of elbow flexion, wrist extension contributes to about a 5.5% increase in NCV at the elbow segment when compared to that with a relaxed wrist position. From an anatomical standpoint this could be explained by the fact that when the wrist is extended, the entire ulnar nerve is stretched further. However, the increase in NCV at the elbow segment is about 3.2% at 90° elbow flexion and about 0.56% at 135°elbow flexion, indicating a reduction in the effect of wrist extension. Since the elbow segment of the ulnar nerve is already stretched to some extent at 90° and to a greater extent at 135° elbow flexion, it seems logical that the contribution of additional stretching due to wrist extension goes down. With wrist flexion, the contribution to nerve stretch at elbow may not occur anatomically, so almost no change was observed in the NCV of the elbow segment.

In the second part of the study, a mathematical tool based on the new nerve conduction mechanism [5] was developed to analyze the change in NCV quantitatively with nerve stretch. However, the proposed scenario at the nodal region with three dimensional interdigitated protrusions of Schwann cell processes from both sides of a node presents a formidable challenge in bringing it into a domain amenable to mathematical analysis. In order to provide a simple and straightforward substitute, a completely different approach was proposed in the present work based on which an analytical tool was developed. This simplified model, based on an open node but filled with insulating microscopic structures offers a viable model relating changes in conduction velocity$\;(\frac{\Delta v}{v})$ of a nerve fibre to obtain quantitative assessments of the changes in nodal resistance ($\frac{{\Delta R}_{ne}}{R_{ne}}$) and resistivity ($\frac{{\Delta \rho }_{eff}}{\rho_{eff}}$) with respect to changes in nerve stretch (*s*).

Finally, introducing experimental values of the increase in NCV with stretch obtained in the present work, it was found that for 1% increase in length (i.e., stretch **s* *= 1%), *R*_ne_ decreases by 1.5% while *ρ*_eff_ decreases by 0.5%. These are significant findings from this study which may help in carrying out further work involving this new mechanism of nerve conduction.

It may be of interest to analyse the results of a classical study on the median giant fiber of Earthworm [27] on stretch which have myelinations and nodal gaps as in humans, but not exactly the same [28,29]. However, the mechanisms of saltatory conduction should be similar. The study showed that between elongations of 1.3 and 2.3 times of the original length of the nerve fibre (both approximate values, as obtained from the given graph), the CV systematically increased almost linearly, much like the linear increase observed on human nerves earlier [20] and in the current study too, although the range of the stretching are far greater than possible in human nerves. Between 2.3 and 4 times of the original length there was no change in CV. One particular issue was that at elongations less than 1.3 times the original length, the researchers did not provide any data. It was mentioned [27] that earthworm nerves have structural folds that allow for elongation and contraction during movement. It is probable that in the initial range of elongation, under 1.3 times the original length, the unfolding of these structures gave random values of CV and therefore, the researchers could not find any systematic variation. The publication [27] also referred to several early papers working on the study of CV with stretch on nerve fibres of annelids, gastropods and leech. Some of these found CV to be constant with stretch while some found an increase in CV. The only mechanism known at that time governing CV in fibres was the combined effect of resistance of the axon and the capacitance of the axonal membrane, offered by Hodgkin and Huxley [9] and the above results contradicted explanations based on these mechanisms. Later, smoothening of folds in the nerve membrane or uncoiling of nerve fibres were proposed by a few authors as reported by Goldman [27] but these were not verified experimentally, nor these could explain all the experimental observations.

Beyond 1.3 times elongation, the folds possibly disappeared, and the nerve then went through stretch on further elongation. The widening of the gaps between the interdigitated Schwann cell processes at the nodal junction, as hypothesized for human nerves [5], caused a gradual decrease in resistance to ionic flow (*R*_*ne*_) which in turn led to the almost linear increase in CV with stretch. Therefore, this is the region where the experimental study on human nerves, which was done to about 30% of stretch, seems to correspond to.

Between 2.3 to 4 times elongations, the NCV was constant even though the resistance was supposed to drop further. This could be due to several factors. One could be that *R*_*ne*_ becomes very low and the inequality of time constants *τ*_*n*_ and *τ*_*m*_ as in Eq. 3 no longer holds so that both contributions are significant but varying over the range. Having opposing effects on NCV due to stretch these time constants could have counterbalanced each other giving rise to a net constant NCV. Secondly, an intuitive suggestion may be put forward, which obviously needs further validation, that the ionic current was possibly limited by the number of ions available for saltatory conduction at each node. So it is the current limit that possibly restrained the CV to a constant value beyond this elongation in conjunction with the nodal capacitance, which is expected to change slightly on stretch. This obviously needs further studies where the ion channels and other microstructures at the node will also be involved. The fall in CV beyond 4 times original length could be due to damage to the nerve at such great pulls, as was also suggested by the authors [27].

Another experimental work [30] which studied CVs for both unmyelinated and myelinated nerves on dissected rat sciatic nerves in-vitro would be of immense interest to interpret based on the new mechanism [5]. Through a very carefully designed experimental work on both myelinated A-fibres and unmyelinated C-fibres of a rat nerve this work found that the percentage increase of delay in relation to the percentage increase in nerve length in A-fibres was almost half that in C-fibres. However, the authors had mistakenly interpreted this as decrease of conduction velocity (CV) for both which were corrected by the authors of the present work in their earlier publication [20]. The corrected scenario was as follows. For a 1% increase in nerve length, the CV in myelinated A-fibres increased by 0.15%, while it decreased by 0.58% for the unmyelinated C-fibres. The latter is what one would expect based on the classical concepts of neuroscience as also mentioned before. However, the systematic increase in CV of myelinated nerve can only be explained by the new mechanism proposed by Rabbani [5] so far.

Although the suggested mechanism implies that structural nodal modifications, particularly of the gaps between interdigitated Schwann cell processes from two sides of a node cause the stretch-induced decrease in *R*_*ne*_ which in turn contribute to the increase in CV, this theory needs to be scrutinized further in view of mechanisms that may still be unknown due to the special structures of Ranvier nodes. The nodal and paranodal regions of myelinated axons have a highly ordered architecture, including glial microvilli, paranodal loops, and cytoskeletal scaffolds like ankyrin-G and βIV-spectrin, according to structural evidence from electron microscopy and molecular studies [25,31]. These elements form tight junctions and maintain ion channel domains. Although these structures seem stable in normal circumstances, no experimental description has been made of how they behave when subjected to mechanical stretch.

Loosening or detachment of paranodal myelin loops and widened paranodal junctional cleft [31] may contribute to reduced paranodal resistance, which may influence conduction by opening a parallel pathway for current around the node (partial short-circuit of paranodal seal). By expanding the nodal extracellular space and eliminating physical barriers to current flow, the retraction or lateral displacement of Schwann cell microvilli from the nodal gap, where structural alterations at the node influence electrophysiological, may reduce resistance to ion flow [32] thus acting in conjugation to interdigitated Schwann cell processes at the nodal junction [5] to decrease the overall nodal resistance (*R*_*ne*_). Therefore, it could be a combined effect, not only that due to increasing gap between Schwann cell (neurilemma) processes. However, further work is necessary to establish this suggestion.

Since both *R*_*ne*_ and *ρ*_*eff*_ are newly introduced parameters, there is no way to verify these with values from others sources. In the future, the findings could be improved through more meticulous, exacting, and rigorous study methods. With the addition of these new mechanisms and parameter characteristics, many previously estimated parameters for myelinated nerves will need re-evaluation, opening up the avenue to a vast amount of work for researchers. Other mechanisms, such as cytoskeletal dynamics, ion channel function at the node or paranode, or stretch-induced changes in membrane tension, could possibly account for the observed results, and need to be evaluated with care. Further ultrastructural and electrophysiological studies may be required to determine whether mechanical deformation of the nodal/paranodal complex actually causes observable changes in resistance and conduction velocity under stretch.
